# A hierarchical lateral stability control strategy of distributed drive electric vehicles based on extended Kalman filter and integral terminal sliding mode control

**DOI:** 10.1371/journal.pone.0341354

**Published:** 2026-02-12

**Authors:** Junzhu Wang, Youqun Zhao, Wei Gao, Zhaowen Deng

**Affiliations:** 1 College of Energy and Power, Nanjing University of Aeronautics and Astronautics, Nanjing, China; 2 School of Intelligent Connected Vehicle, Hubei University of Automotive Technology, Shiyan, China; Beijing Institute of Technology, CHINA

## Abstract

This paper proposes a hierarchical control strategy to enhance the lateral stability of distributed drive electric vehicles. In the upper layer, the extended kalman filter (EKF) is employed for real-time estimation of critical vehicle states, including the sideslip angle and yaw rate. In the intermediate layer, a direct yaw-moment control (DYC) system based on integral terminal sliding mode control (ITSMC) is designed, which utilizes the deviation between the EKF-estimated states and their desired values to calculate the required additional yaw moment for stability compensation. In the lower layer, an optimal control–based torque allocation strategy is adopted to distribute the driving torque among the four in-wheel motors. Unlike many existing direct yaw moment control strategies that assume ideal state availability or suffer from control chattering and limited wheel-level realizability, this study explicitly addresses the coupled problem of state estimation uncertainty, robust yaw-moment generation, and practical torque realization under nonlinear tire dynamics. Simulation results demonstrate that the proposed EKF-based state estimation achieves high accuracy, while the ITSMC-DYC controller significantly improves lateral stability, trajectory tracking capability, and driving safety. Furthermore, hardware-in-the-loop (HIL) tests validate the effectiveness of the hierarchical control strategy under realistic scenarios, confirming its potential for practical applications.

## 1. Introduction

The development of the automotive industry and the continuous advancement of motor and battery technology has led to a focus on electric vehicles due to the advantages of energy saving and environmental protection. Distributed drive electric vehicles (DDEVs) have the unique advantages of independently controllable drive torque and braking torque, high control precision, and high transmission efficiency, etc [1]. Therefore, DDEVs are easier to realize vehicle dynamics control, and have a promising future in the electric vehicle industry [2]. However, the same distributed and highly coupled actuation characteristics that provide superior controllability also introduce new challenges to vehicle lateral stability, especially under nonlinear tire behaviors and strong coupling between longitudinal and lateral dynamics.

It is undeniable that the lateral stability of DDEVs is poor than that of the traditional internal combustion engine vehicles, especially when DDEVs drive under high-g maneuvers or on slippery roads. This degradation is mainly caused by the rapid variation of tire forces, uneven vertical load transfer, and the absence of mechanical coupling between wheels, which amplifies sensitivity to modeling uncertainties and road disturbances. To avoid the occurrence of the potential accident, various active safety systems, such as anti-lock braking system [3], active suspension control system [4], etc., have been developed for improving the lateral stability and safety of the DDEVs under emergency scenarios. But the control effect of these active safety systems mainly depends on the accurate vehicle states, including side slip angle of the center of gravity, yaw rate, longitudinal vehicle speed, etc. Unfortunately, not all the knowledge of vehicle states can be directly measured for mass-production vehicles due to the high cost or the technique constraints of the sensors.

Therefore, a lot of research has been carried out on vehicle state estimation technique to obtain accurate and reliable vehicle states information for active safety systems. Kalman filter theory has been widely used in many engineering fields, especially in navigation system [5], which is also employed to estimate vehicle states in the automobile field. However, the standard Kalman filter is only suitable for linear systems. Among various nonlinear filtering approaches, the Extended Kalman Filter (EKF) has become a predominant solution due to its relatively simple structure and computational efficiency for moderately nonlinear systems like vehicle dynamics [6–9]. Jiang et al. [6] adopt the extended Kalman filter with a fusion algorithm to estimate the side slip angle and the roll angle of a vehicle. Simulations and real-car tests shows that the estimation method has good robustness and effectiveness. The performance of the extended Kalman filter and unscented Kalman filter was compared by real vehicle test [7], with results indicating comparable accuracy but higher efficiency for the EKF. However, the effectiveness of model-based estimators like EKF is inherently contingent upon the accuracy of the underlying vehicle and tire models. In real-world driving, model uncertainties, varying road conditions, and sensor noise pose persistent challenges. To address these, some studies have focused on joint estimation of states and parameters [10,11,12], or integrating intelligent algorithms with EKF [13,12]. A two-stage method for vehicle sideslip angle and tire parameters estimation is proposed and validated by real car experiment [10]. The vehicle states and tire forces or stiffness are estimated jointly by using extended Kalman filter in the first real-time stage. Yi et al. presents a vehicle states estimation method combining genetic algorithm and extended Kalman filter [13]. Compared with the standard extended Kalman filter, the method improves the accuracy of vehicle states estimation. A model-based vehicle states and parameters combined estimator was designed using the dual extended Kalman filer technique [11]. The effectiveness of the method was verified, and the potential benefits was analyzed. Reina et al. design an observer to jointly estimate the vehicle states and tyre cornering stiffness by using an extended Kalman filter framework [12]. A novel fuzzy adaptive fault-tolerant extended Kalman filter is proposed, in which a fault-tolerant mechanism is integrated to handle missing measurements and a fuzzy logic system is employed to dynamically adjust the process noise covariance matrix, with its superior estimation accuracy under various driving conditions and data loss scenarios being validated by extensive experiments [14]. Although these methods demonstrate good estimation performance, most of them focus primarily on improving estimation accuracy itself, without explicitly analyzing how estimation uncertainty propagates into stability control performance under extreme driving conditions. In addition, many advanced estimators rely on complex structures or additional tuning parameters, which may limit their real-time applicability and robustness when integrated with nonlinear controllers in DDEVs.

To improve the lateral stability of the vehicle, the commonly used control methods are active steering control (ASC) and direct yaw-moment control [15]. Compared with ASC, DYC has better ability to maintain vehicle stability by utilizing the additional yaw moment, especially on high lateral acceleration maneuver or slippery roads. Moreover, the additional yaw moment used to improve vehicle stability is generated when the driving or braking forces between the left and right wheels are different [16–18]. Because the DDEVs have the advantages of in-wheel motor torque independent controllable, therefore, in comparison with traditional internal combustion cars, DYC can be more easily and flexibly implemented on DDEVs [19, 20]. Many control algorithms for DYC systems have been devised to improve the lateral stability of DDEVs, such as sliding mode control (SMC) [20, 21], Model Predictive Control (MPC) [22], nonlinear model predictive control (NMPC) [23], reinforcement learning-based [24], etc. A robust integrated motion control framework is proposed for CAV platoons, where a fuzzy PID-based longitudinal controller and an MPC-based lateral controller are first developed for individual vehicles, and subsequently a robust H-infinity longitudinal controller integrated with an APF-based MPC lateral controller is established for the entire platoon, with the effectiveness and limitations of these controllers being validated through comprehensive simulations [25]. However, existing DYC methods often suffer from at least one of the following limitations: (i) strong dependence on accurate vehicle and tire models (e.g., MPC-based approaches), (ii) chattering and actuator wear induced by discontinuous control laws (e.g., conventional SMC), or (iii) lack of explicit robustness guarantees under rapidly varying tire-road conditions.

In distributed electric drive vehicles, the reasonable distribution of direct yaw torque among four in-wheel motors is critical for maximizing stability margins [26, 27]. Current research focuses mainly on optimizing yaw moment generation and torque distribution strategies [28–31]. Semaan A et al. designed an artificial neural network optimization algorithm, according to the neural network principle, to estimate the direct yaw torque required to maintain yaw stability in a distributed electric drive vehicle [29]. Chen et al proposed a hub motor torque distribution strategy, based on model predictive control, for application to distributed drive electric vehicles, implementing an energy efficiency-based strategy and a minimum power loss-based strategy for hub motor torque distribution, respectively [30]. Ningyuan Guo et al. used a CarSim embedded drive model, and a DYC controller based on a linear quadratic regulator (LQR), as virtual inputs to calculate the required longitudinal traction force and yaw moment, respectively, to optimize tire slip rate and vehicle stability [31]. Nevertheless, in many existing studies, the upper-layer yaw moment controller and the lower-layer torque distribution algorithm are designed independently, and the interaction between estimation accuracy, yaw moment robustness, and torque allocation under load transfer is rarely discussed in an integrated framework. Moreover, the majority of these works implicitly assume that sideslip angle and yaw rate are readily available or perfectly estimated, which may not hold in real vehicles operating near handling limits.

The above studies of vehicle stability control have used the parameters of the side slip angle and yaw rate, but the methods of obtaining these two signals have not been specified. In many cases, these key state variables are implicitly assumed to be measurable or ideal, which limits the practical applicability of the proposed control strategies when deployed on real vehicles operating near handling limits. To enhance the accuracy of vehicle parameter state estimation, the control algorithm is usually complicated, but it will have certain disadvantages [32]. Specifically, there exists a clear trade-off between estimation accuracy, computational complexity, and real-time feasibility in engineering applications. For example, the common sliding mode observation method causes inaccurate estimation, due to its jitters. The particle filtering algorithm is poor in real-time, and the fuzzy rules used by the fuzzy logic method rely on experience in use. These limitations indicate that although advanced estimation techniques may improve accuracy under specific conditions, their robustness and scalability remain insufficient for integrated stability control of distributed drive electric vehicles.

Therefore, under the premise of ensuring that the vehicle driving state accuracy requirements are met, this paper estimated the state of the vehicle’s side slip angle and yaw rate, based on the extended Kalman Filter algorithm. The EKF provides a balanced solution by combining acceptable estimation accuracy with moderate computational cost, making it suitable for real-time implementation and subsequent controller integration. The sliding mode control method has been proved to be a control method that is robust to parameter changes and unknown external disturbances, which has good tracking performance [33]. However, in practical engineering, the accuracy of the nonlinear model of the system is difficult to guarantee, and the chattering phenomenon caused by the sliding surface is inevitable. This chattering not only degrades control smoothness but may also induce actuator wear and deteriorate yaw moment realization in DDEVs. In literature [34], considering the uncertainty of parameters, an integral terminal sliding mode controller was proposed. The controller can quickly achieve lateral stability and effectively reduce chattering. To alleviate the chattering of the controller to the greatest extent, it is necessary to adopt the SMC controller combining the integral term and the terminal term to control the yaw stability of the vehicle.

In summary, existing studies on lateral stability control of distributed drive electric vehicles still face several unresolved challenges, including the implicit assumption of full-state availability, limited robustness of yaw-moment controllers under strong nonlinearities, and insufficient consideration of wheel-level realizability under dynamic load transfer. To address these issues, this paper proposes a hierarchical lateral stability control strategy for distributed drive electric vehicles. The main contributions of this study can be summarized as follows:

(1) A tightly coupled EKF-based vehicle state estimation and control framework is established, in which the estimated sideslip angle of the center of gravity and yaw rate are directly used as feedback signals for lateral stability control, avoiding the common assumption of directly measurable ideal states and enhancing practical applicability under extreme driving conditions.
(2) An integral terminal sliding mode control–based direct yaw moment controller is developed to improve robustness against parameter uncertainties and external disturbances, while effectively suppressing chattering and ensuring finite-time convergence of stability-related states, making it suitable for distributed drive electric vehicles operating near handling limits.
(3) A hierarchical implementation scheme integrating the upper-layer yaw-moment control with a lower-layer optimal control–based wheel torque allocation strategy is proposed, in which the torque distribution problem is formulated as an optimization task under actuator and tire–road constraints, ensuring that the yaw-moment demand can be effectively and efficiently realized at the wheel level. The overall effectiveness and engineering feasibility of the proposed strategy are validated through co-simulation and hardware-in-the-loop tests.

The rest of the paper is organized as follows. Section 2 presents and validates the vehicle model. A hierarchical lateral stability control strategy is designed in Section 3. The simulation results are analyzed in Section 4. The control effectiveness of strategy has been proved by HIL test in Section 5. Finally, conclusions are drawn in Section 6.

## 2. System modelling

### 2.1. 7-DOF nonlinear vehicle dynamics model

**Fig 1:**
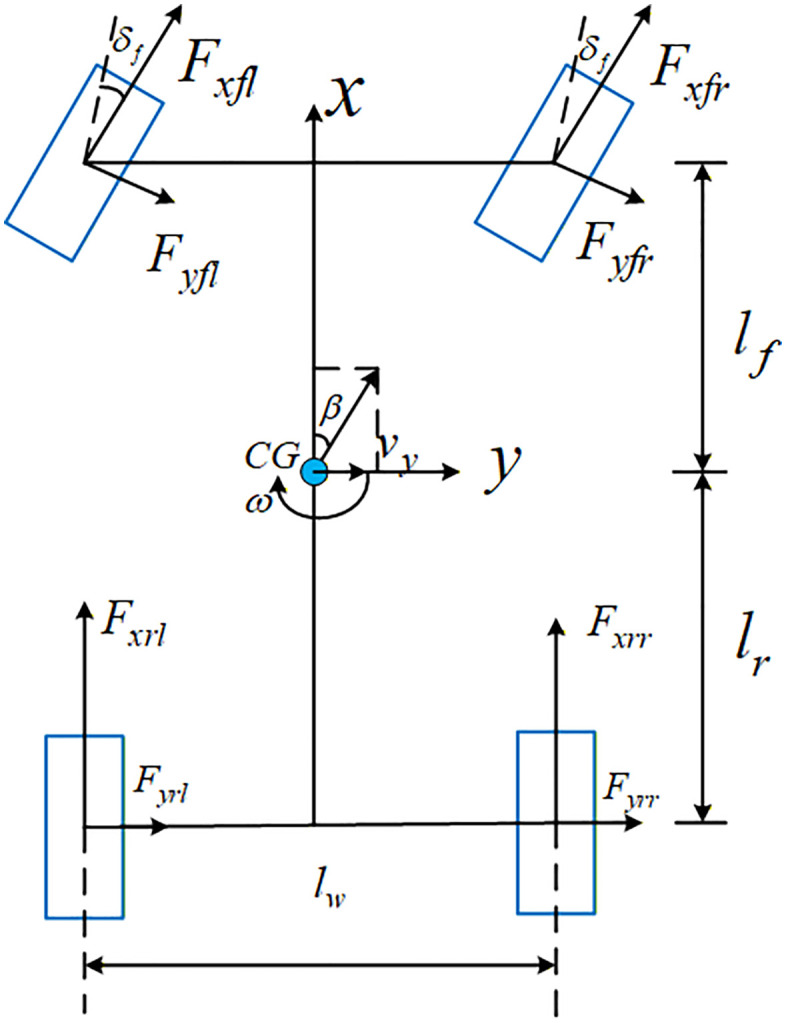
7-DOF nonlinear vehicle model.

The 7-DOF nonlinear vehicle model during steering is shown in Fig 1. Considering the coupling characteristics between the lateral and longitudinal directions of the vehicle, a 7-DOF vehicle model was established. The 7-DOF nonlinear vehicle model is used for controller design, theoretical verification, and algorithm development. This model is transparent in structure and adjustable in parameters, facilitating the integration of model-based control algorithms and helping to reveal the intrinsic mechanisms of vehicle dynamic responses.

The effect of the suspension system and steering system is ignored, and the front wheel turning angle is taken directly as an input to the system; The effect of air resistance on the vehicle model is ignored; The Centre of gravity of the vehicle is taken as the origin of the vehicle coordinate system; The tires are the same for all four wheels.

The lateral and yaw motion differential equations of the vehicle are shown in equation (1).


$$\left\{ \begin{array}{c} cmv_{x}(\dot{\beta }+\omega )=(F_{yfl}+F_{yfr})\cos\delta_{f}+F_{yrl}+F_{yrr}+(F_{xfl}+F_{xfr})\sin\delta_{f}\\ I_{z}\dot{\omega }=a(F_{yfl}+F_{yfr})\cos\delta_{f}-b(F_{yrl}+F_{yrr})+[(F_{xfr}-F_{xfl})\cos\delta_{f}+(F_{yfl}-F_{yfr})\sin\delta_{f}]\frac{l_{w}}{2}+[(F_{xrr}-F_{xrl})]\frac{l_{w}}{2} \end{array}\right.$$ (1)

The longitudinal degree of freedom differential equation of the vehicle is shown in equation (2).


$$m\left({\dot{v}}_{x}+\omega \beta v_{x}\right)=\left(F_{xfr}+F_{xfl}\right)\cos\delta_{f}+\left(F_{yfl}+F_{yfr}\right)\sin\delta_{f}+F_{xrr}+F_{xrl}$$ (2)

The rotational freedom of the wheel is shown in equation (3).


$$I_{w}{\dot{w}}_{i}=T_{di}-F_{xi}R_{w}-T_{bi}\left(i=fl,fr,rl,rr\right)$$ (3)

where, *m* denotes the mass of the vehicle (kg); $v_{x}$ denotes the longitudinal speed of the vehicle (m/s); β denotes the side slip angle of CG (rad); ω is the yaw rate of the vehicle (rad/s); $F_{yfl}$, $F_{yfr}$*,* $F_{yrl}$*,* $F_{yrr}$ denote the lateral force of the left front wheel, right front wheel, left rear wheel and right rear wheel (N), respectively; a,b are the distance from the CG to the front and rear axles (m), respectively; $\delta_{f}$ is the steering angle of the front wheels (rad), and $I_{z}$ is the inertia moment about the Z-axis ($kg\cdot m^{2}$); $I_{w}$ is the rotational inertia of the wheel; $w_{i}$ is the rotational angular velocity of the wheel (rad/s); $T_{di},T_{bi}$ are the driving torque and braking torque of the wheel (N·m), respectively; l is the wheelbase between the front and rear axles(m).

The basic parameters of the vehicle are shown in Table 1. These parameters correspond to a B-class vehicle and are directly adopted from a high-fidelity CarSim vehicle model, which is widely used as a benchmark simulation platform in vehicle dynamics research.

**Table 1:** The basic parameters of the vehicle.

Meaning	Parameter	Value
total mass of vehicle, kg	*m*	1230
distance from the CG to the front axles, m	*a*	1.04
distance from the CG to the rear axles, m	*b*	1.56
inertia moment of mass about the vertical axles, kg·m^2^	*I*_*z*_	1343.1
steering system ratios	$i_{sw}$	25.7
the wheelbase of wheels, m	$l_{w}$	1.48
the effective rolling radius of the wheels, m	$R_{sw}$	0.298

### 2.2. Tire model

**Fig 2:**
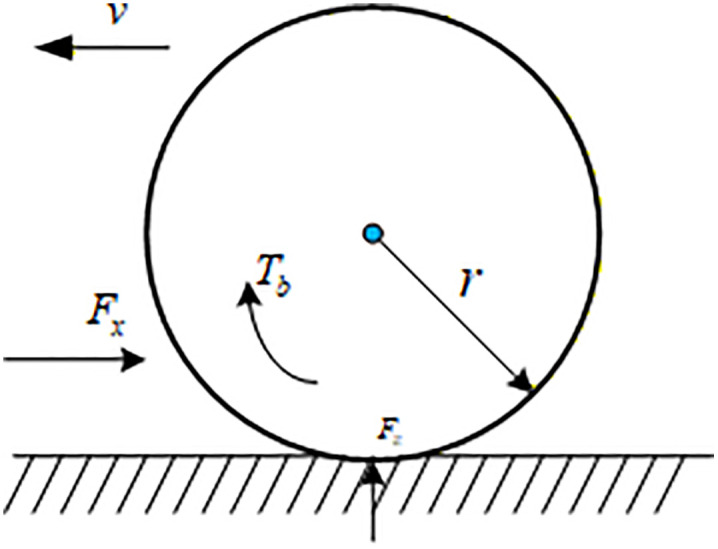
Wheel rotation motion diagram.

The force analysis of the tire is shown in Fig 2. Due to the nonlinearity of the tire, there will be some errors when using linear tires for verification, so the magic tire model Magic Formula is used [11,35].

The general expression of magic tire lateral force is,


$$\left\{ \begin{array}{c} \;F_{y}=y(x)+S_{v}\\ \;y=D\sin\{C\;\arctan[Bx-E(Bx-\arctan(Bx))]\}\\ x=X+S_{h} \end{array}\right.$$ (4)

where, X is the side slip angle of the tires, D is the peak factor, C is the curve shape factor, B is the stiffness factor, E is the curve curvature factor, $S_{h}$ is the drift in the horizontal direction of the curve, $S_{v}$ is the drift in the vertical direction of the curve. Where, C=1.3, $D=a_{1}F_{z}^{2}+a_{2}F_{z}$, $BCD=a_{3}\sin\left[a_{4}arctan\left(a_{5}F_{z}\right)\right]\left(1-a_{12}\left|\gamma \right|\right)$, $B=\frac{BCD}{CD}$, $E=a_{6}F_{z}^{2}+a_{7}F_{z}+a_{8}$, $S_{h}=a_{9}\gamma$, , γ is the camber angle of the wheel.

The general expression of magic tire longitudinal force is.


$$\left\{ \begin{array}{c} \;F_{x}=y(x)\\ \;y=D\sin\{C\arctan[Bx-E(Bx-\mathrm{arctam}(Bx))]\}\\ x=\lambda \end{array}\right.$$ (5)

where C=1. 65, $\;D=a_{1}F_{z}^{2}+a_{2}F_{z}$, $\;BCD=\left(a_{3}F_{z}^{2}+a_{4}F_{z}\right)*e^{-a_{5}F_{z}}$, $\;B=\frac{BCD}{CD}$, $\;E=a_{6}F_{z}^{2}+a_{7}F_{z}+a_{8}$.

The wheel camber angle is the angle between the wheel plane and the vertical axis of the vehicle coordinate axis. Under the ideal condition, the influence of the wheel camber angle and drift is not considered, that is, the tire camber angle γ is 0, $S_{h}$, $\;S_{v}$ are 0, respectively. The fitting parameters are shown in Table 2. These parameters are obtained by fitting the tire force characteristics extracted from the CarSim vehicle model, ensuring consistency between the simplified tire model used for control design and the high-fidelity tire behavior represented in CarSim simulations.

**Table 2:** Magic tire fitting parameters.

	$a_{1}$	$a_{2}$	$a_{3}$	$a_{4}$	$a_{5}$	$a_{6}$	$a_{7}$	$a_{8}$	$a_{9}$	$a_{10}$	$a_{11}$	$a_{12}$
$F_{y}$	-22.1	1011	1078	1.82	0.208	0	−0.354	0.707	0.028	0	14.8	0.022
$F_{x}$	-21.3	1144	49.6	226	0.069	−0.006	0.058	0.486				

### 2.3. Ideal state model

To design the direct yaw moment controller, the ideal yaw rate and sideslip angle of the vehicle are introduced as reference states. These ideal states are derived based on the steady-state response of the linear bicycle model under small-angle assumptions, which has been widely adopted in classical vehicle dynamics studies for lateral stability analysis.

By assuming steady-state cornering conditions, i.e.,


$${\dot{v}}_{y}=0,\dot{\omega }=0$$ (6)

the desired yaw rate can be derived from the linear two-degree-of-freedom vehicle model as [36]:


$$\omega_{d}=\frac{v_{x}/L}{1+K_{s}{v_{x}}^{2}}\delta_{f}$$ (7)

where the stability factor $K_{s}$ is defined as $K_{s}=\frac{m}{L^{2}}(\frac{a}{k_{r}}-\frac{b}{k_{f}})$.This formulation is consistent with classical steady-state yaw rate expressions reported in fundamental vehicle dynamics literature.

Considering the limitation imposed by tire–road adhesion, the ideal yaw rate is subject to an upper bound, which can be expressed as:


$$\omega_{m}=\frac{\mu g}{v_{x}}$$ (8)

where *µ* is the road adhesion coefficient and *g* denotes the gravitational acceleration. Therefore, the ideal yaw rate is finally defined as:


$$\omega_{d}=\{\begin{array}{c} \frac{v_{x}/L}{1+K_{s}{v_{x}}^{2}}\delta_{f},\;|\omega |<\omega_{m}\\ \omega_{m}\mathrm{sign}(\omega ),\;|\omega |\geq \omega_{m} \end{array}$$ (9)

which ensures that the reference yaw rate remains within physically feasible limits.

For the sideslip angle, extensive vehicle dynamics studies have shown that maintaining a small sideslip angle is a key indicator of lateral stability. Therefore, the ideal sideslip angle is defined as:


$$\beta_{d}=0$$ (10)

This choice implies that the control objective is to suppress excessive lateral motion and guide the vehicle toward a stable state without significant sideslip. The above ideal state model, consisting of a bounded yaw rate reference and zero sideslip angle reference, has been widely adopted in yaw stability and direct yaw moment control design based on classical vehicle dynamics theory. These ideal states serve as reference inputs for the proposed controller and provide a clear and physically meaningful stability target.

### 2.4. Motor model

Since the focus of this paper is on the control of the lateral stability of the vehicle, the motor model is simplified to some extent in the process of building the motor model, disregarding the effects of inductance and damping, and only the relationship between the target torque of the motor and the actual torque is obtained. The relationship can be simplified to the form of a two-order transfer function.


$$G(s)=\frac{T_{r}}{T_{d}}=\frac{\text{1}}{2\xi^{2}s^{2}+2\xi s+1}$$ (11)

where, $T_{r}$ is the actual output torque of the tires, ξ is the motor characteristic parameter, the paper takes 0.01. $T_{d}$ is the target torque required by the motor.

### 2.5. Vehicle dynamics model validation

**Fig 3:**
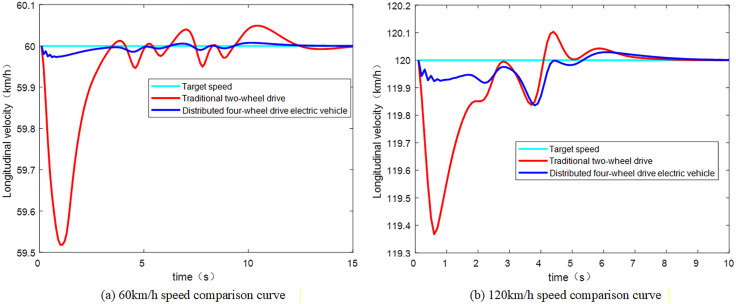
Speed comparison curve.

To verify the effectiveness of the constructed four-motor drive system, a comparison was made with the front-wheel-drive vehicle in CarSim to verify the speed tracking capability at 60 km/h and 120 km/h, respectively. The speed tracking comparison results are shown in Fig 3.

As can be seen from Fig 3, the speed error of the DDEV is controlled by 0.1%, and that of the conventional two-wheel drive vehicle with an internal combustion engine is controlled by 0.8%, under both high-speed and low-speed conditions, both of which meet the experimental requirements. The response to speed changes of the DDEV is better than that of the conventional two-wheel drive vehicle, due to the independent drive of the four wheels.

## 3. Hierarchical lateral stability control strategy design

### 3.1. Upper-layer state estimation system design

Accurate estimation of vehicle states is fundamental to the control strategy [37]. This section will use EKF to achieve accurate estimation of the vehicle’s state. The Extended Kalman Filter was chosen based on a comprehensive assessment of nonlinear estimators concerning accuracy, computational load, and output characteristics. Compared to the Unscented Kalman Filter, the EKF offers superior computational efficiency while meeting the required estimation accuracy, making it more suitable for real-time implementation. The Particle Filter is deemed unsuitable for high-frequency estimation due to its high computational complexity and sample degeneracy issues. Although Sliding Mode Observers are robust, their outputs exhibit inherent chattering, which is undesirable for generating smooth control commands. Consequently, the EKF is selected for this study due to its smooth optimal estimates, moderate computational cost, and proven engineering applicability [38,39].

The design objectives of the EKF in this study include not only high-precision estimation of the immeasurable sideslip angle but also filtering optimization of the yaw rate, along with ensuring state consistency through dual-state synchronous estimation. Furthermore, the EKF provides a degree of sensor fault tolerance. In the event of yaw rate sensor anomalies, it can maintain continuous state estimation based on the vehicle model and other available signals.

The state observation equations of the EKF are constructed using their dynamics equations, in conjunction with the established 2-DOF vehicle model. The equations of state and the observation equations are as follows.


$$\dot{x}(t)=f\left(x(t),u(t),w(t)\right)$$ (12)

y(t)=h(x(t),v(t)) (13)

where, x(t) is the state variable, u(t) is the control variable, y(t) is the measured output of the system, w(t),v(t) are the process noise and the measurement noise, respectively.

The 2-DOF model is brought into the state and observation equations to reestablish the state equations of the system.


$$\left\{ \begin{array}{c} \dot{\beta }=\frac{k_{f}+k_{r}}{mv_{x}}\beta +(\frac{ak_{f}-bk_{r}}{mv_{x}^{2}}-1)\omega -\frac{k_{f}}{mv_{x}}\delta_{f}\\ \dot{\omega }=\frac{ak_{f}-bk_{r}}{I_{z}}\beta +\frac{a^{2}k_{f}+b^{2}k_{r}}{I_{z}v_{x}}\omega -\frac{ak_{f}}{I_{z}}\delta_{f} \end{array}\right.$$ (14)

The observation equation for the system is given by,


$$a_{y}=\frac{K_{f}+k_{r}}{m}\beta +\frac{a*K_{f}-b*K_{r}}{mv_{x}}\omega -\frac{K_{f}}{m}\delta_{f}$$ (15)

where, $\dot{x}(t)={\left[\dot{\beta }\;\dot{\omega }\right]}^{T}$, $y(t)={\left[a_{y}\;\omega \right]}^{T}$.

The model was linearized, and let F(t),H(t) be the Jacobi matrix of f,h for the partial derivatives of x(t), respectively. Δt is the sampling time.


$$F(t)={\frac{\partial f(x)}{\partial x}|}_{x=x(t)}$$ (16)

$$H(t)={\frac{\partial h(x)}{\partial x}|}_{x=x(t)}$$ (17)

$$\dot{x}(t)=\frac{x(t+1)-x(t)}{\Delta t}$$ (18)

Substituting equation (16) into equation (18).


x(t+1)=(I+F(t)·Δt)·x(t) (19)

$$F(t)=[\begin{array}{cc} \frac{K_{f}+K_{r}}{mv_{x}} & \frac{aK_{f}-bK_{r}}{mv_{x}^{2}}-1\\ \frac{a*K_{f}-b*K_{r}}{I_{z}} & \frac{a^{2}K_{f}+b^{2}K_{r}}{I_{z}v_{x}} \end{array}]$$

$$H(t)=[\begin{array}{cc} \frac{K_{f}+K_{r}}{m} & \frac{aK_{f}+bK_{r}}{mv_{x}}\\ 0 & 1 \end{array}]$$

**Fig 4:**
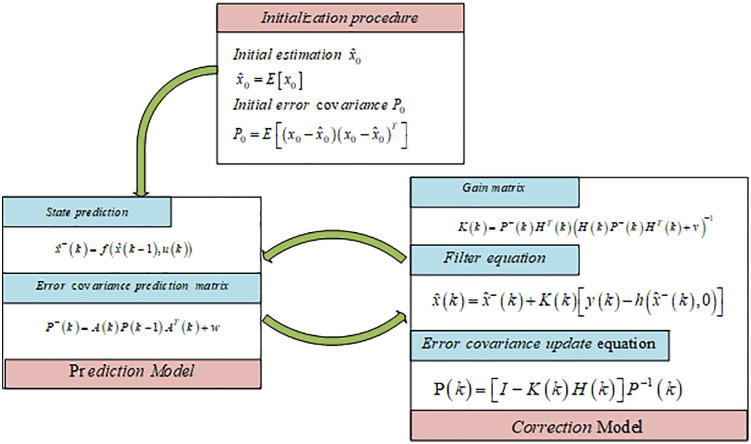
The EKF workflow.

The EKF workflow is shown in Fig 4 [16,17,40]. k is the time step, ${\hat{x}}^{-}\left(k\right)$ is the prior estimate of the prediction using the previous state variable, $\overset{̑}{x}(k)$ is the posterior estimate of the system state variables at moment k, ${\hat{x}}_{\text{0}}$ is the initial value of state estimation, $P_{\text{0}}$ is the initial covariance matrix of state estimation, u(k) is the control variable, w is the process noise, $P^{-}\left(k\right)$ is the prior covariance matrix at moment k, Q,R are the covariance matrices of the process noise and measurement noise, respectively, K(k) is the Kalman gain, y(k) is the measurement variable, A is the state transfer matrix, B is the control matrix, H is the observation matrix. Through the iterative operation of the prediction and correction equations, the prior results of the state estimation at moment k can be obtained from the estimates of the state variables at moment k −1, and then the posterior results of the state estimation at the moment k of the Kalman Filter are updated by the measurement results at the moment k.

For nonlinear systems, the EKF expands the first-order Taylor of the system, ignoring the second-order and other high-order terms, which will have a certain impact on the accuracy of the model. However, in the automobile steering system, the tire can be approximated as a linear system in a certain area, so the EKF algorithm can obtain higher estimation accuracy.

### 3.2. Middle-layer direct yaw moment control system design

**Fig 5:**
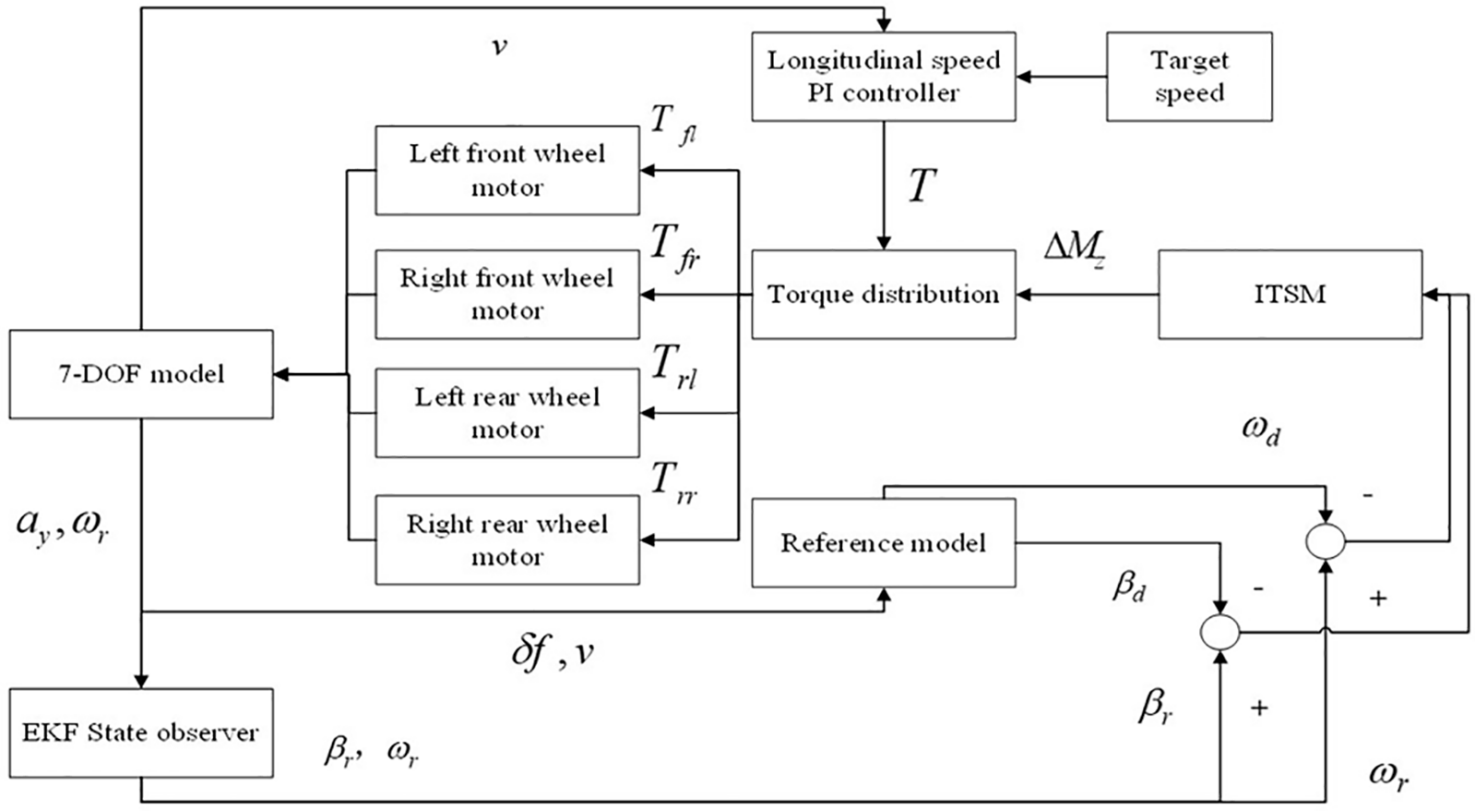
ITSMC control system.

To improve the driving stability of the vehicle, this paper proposes an ITSMC algorithm to design the DYC controller. By controlling the vehicle’s side slip angle and yaw rate to follow the desired values, the vehicle has good maneuvering stability under a variety of operating conditions. The Direct Yaw Moment Controller must ensure stability under model uncertainties and external disturbances. Integral Terminal Sliding Mode Control (ITSMC) was chosen for its ability to effectively balance robustness, response speed, and engineering feasibility. Compared to conventional Sliding Mode Control, ITSMC significantly suppresses detrimental control chattering through the incorporation of an integral term and a saturation function. The performance of Model Predictive Control heavily relies on an accurate prediction model, posing significant challenges under extreme operating conditions with severe parameter variations. Methods such as H∞ control may involve complex designs and do not guarantee finite-time convergence. ITSMC combines the strong robustness of the integral term, the finite-time convergence property of the terminal term, and smooth control output, making it more suitable for the rapid stabilization requirements of this application.The structure of the control system is shown in Fig 5. The 7-DOF nonlinear vehicle model was chosen as the actual vehicle model, the state observer was built based on the extended Kalman filter algorithm, and the established 2-DOF linear vehicle model was adopted as the reference model. The actual lateral acceleration $a_{y}$ and the actual yaw rate ω of the vehicle are output by the 7-DOF nonlinear model, after observation by the EKF state observer, the side slip angle $\beta_{r}$ and the actual yaw rate ω are output. At the same time, the 7-DOF nonlinear model outputs the front wheel angle $\delta_{f}$ and the longitudinal speed of the vehicle v to the reference model, and calculates the ideal side slip angle $\beta_{d}$ and yaw rate $\omega_{d}$,which are input to the ITSMC controller together with the real signal obtained by the EKF state observer. Through control of the ITSMC controller, the required additional yaw moment for the whole vehicle $\Delta M_{z}$ is obtained. After receiving the actual vehicle speed from 7-DOF vehicle model and the target speed, the longitudinal speed PI controller calculates the whole drive torque T required to track the target vehicle speed, the stable control of the driving speed is realized and the desired longitudinal force is obtained. The whole drive torque output from the longitudinal speed PI controller and the whole additional yaw moment output from the ITSMC is transmitted to the lower torque distribution controller, and then the torque is distributed based on the dynamic load distribution method, to obtain the drive torque of the four drive wheels $T_{fl},T_{fr},T_{rl},T_{rr}$, and then the drive torque is input to the 7-DOF vehicle model and transmitted to the drive motors, finally, the closed-loop control of vehicle stability is realized.

With the addition of the additional yaw moment, the 2-DOF model of the vehicle is:


$$\left\{ \begin{array}{c} \dot{\beta }=\frac{k_{f}+k_{r}}{mv_{x}}\beta +(\frac{ak_{f}-bk_{r}}{mv_{x}^{2}}-1)\omega -\frac{k_{f}}{mv_{x}}\delta_{f}\\ \dot{\omega }=\frac{ak_{f}-bk_{r}}{I_{z}}\beta +\frac{a^{2}k_{f}+b^{2}k_{r}}{I_{z}v_{x}}\omega -\frac{ak_{f}}{I_{z}}\delta_{f}+\frac{1}{I_{z}}\Delta M \end{array}\right.$$ (20)

According to equation (20), the relative relationship between the additional yaw moment *ΔM* and yaw angular acceleration $\dot{\omega }$ is as follows,


$$\dot{\omega }=f+d*\Delta M$$ (21)

Among them, f and d can write the combination of certain items and uncertain items, respectively.


$$\left\{ \begin{array}{c} f=\hat{f}+\Delta f\in [f_{\min},f_{\max}]\\ d=\hat{d}+\Delta d\in [d_{\min},d_{\max}] \end{array}\right.$$ (22)

In equation (22), when $\hat{f}=0$ and $\hat{d}=\frac{\text{1}}{I_{z}}$, $\hat{d}$ is a constant, and the uncertainty of the system satisfies the matching condition. Therefore, when the uncertainty and control input are in the same system channel, the influence of uncertainty can be weakened by certain control measures.

It is assumed that the deviation of uncertainty is bounded, that is:


$$\left\{ \begin{array}{c} |\Delta f|=|f-\hat{f}|\leq F\\ 0\leq \rho^{-1}\leq d*{\hat{d}}^{-1}\leq \rho \end{array}\right.$$ (23)

where, *F* > 0, $\rho ={\left(\frac{d_{\max}}{d_{\min}}\right)}^{\frac{\text{1}}{\text{2}}}$.

The aforementioned boundedness assumption (Eq. (23)) provides a theoretical foundation for the controller to handle uncertainties or perturbations in practical system parameters, such as tire cornering stiffness. The objective of the controller design is precisely to ensure that the system remains stable under such bounded uncertainties.

The control variable deviation is [41,42]:


$$e=k_{1}(\beta -\beta_{d})+k_{2}(\omega -\omega_{d})$$ (24)

where *k*_1_ and *k*_2_ are the weighting coefficients.

According to the design method of Sliding mode surface and the characteristics of vehicle stability control system, the Integral Terminal Slide Mode (ITSM) controller is selected, the integral term can solve the external disturbance and effectively improve the robustness of the system, the lateral stability of the vehicle can be enhanced by obtaining the desired yaw moment [18]. The terminal term is exponential, which ensures the convergence of the system. In order to make the actual yaw rate and side slip angle track the ideal value, the Sliding mode surface is designed as follows [19,20].


$$s=e+\gamma \int_{0}^{t}e^{q/p}(\tau )d\tau$$ (25)

where γ>0 is design parameter, p>q>0 are odd numbers, ensuring finite-time convergence (q/p<1). The derivative of s is calculated as:


s˙=e˙+γeq/ps\u02D9=k1(β˙−β˙d)+k2(ω˙−ω˙d)+γeq/ps\u02D9=k1(β˙−β˙d)+k2((f+d*ΔM)−ω˙d)+γeq/p (26)

When $\dot{S}=0$ the equivalent process control input can be solved as,


$$\Delta \hat{M}=-\frac{1}{k_{2}\hat{d}}(k_{2}f-k_{2}{\dot{\omega }}_{d}+k_{1}(\dot{\beta }-{\dot{\beta }}_{d})+\gamma e^{q/p})$$ (27)

In order to achieve dynamic synovial membrane, the exponential approach rate is used,


$$\dot{s}=-\varepsilon sgn(s)-\sigma (s)$$ (28)

where, ε is the thickness of the boundary layer and σ is the convergence rate index.

The output variable is U=[ΔM], combining equations (26), (27) and (28) yields the output variable as,


$$\Delta M=-\frac{1}{k_{2}\hat{d}}(k_{2}f-k_{2}{\dot{\omega }}_{d}+k_{1}(\dot{\beta }-{\dot{\beta }}_{d})+\gamma e^{q/p})-\varepsilon \mathrm{sgn}(s)-\sigma (s)$$ (29)

To eliminate any chattering, a continuous function sat(S) is used instead sgn(S).


$$\mathrm{sat}(S)=\{\begin{array}{c} \;1,\;S>\Delta \\ \;\lambda S,|S|\leq \Delta ,\Delta =\frac{1}{\lambda }\\ -1,\;x<\Delta \end{array}$$ (30)

The design parameters $k_{1}$ and $k_{2}$ mainly affect the convergence speed and robustness of the sliding dynamics, while the parameters p and q determine the nonlinear structure of the terminal sliding surface and influence the finite-time convergence behavior. The parameter γ controls the switching intensity of the sliding mode term and plays a key role in balancing robustness and chattering suppression.

According to the Lyapunov second method in modern control theory, defining a scalar function with positive definiteness V(x), and because $\dot{V}\left(x\right)$ is negative definite, the system is asymptotically stable. Define the Lyapunov function as.


$$V=\frac{\text{1}}{\text{2}}S^{2}$$ (31)

Derivation of equation (31) yields,


$$\dot{V}=\dot{S}*S=-\varepsilon S*sign(S)-\sigma S^{2}$$ (32)

$$\dot{V}=\{\begin{array}{c} \dot{S}*S=-\varepsilon S-\sigma S^{2},\;S>0\\ \dot{S}*S=0,\;S=0\\ \dot{S}*S=\varepsilon S-\sigma S^{2},\;S<0 \end{array}$$ (33)

Duo to $\dot{V}\leq 0$, it is known to indicate that the designed system is stable.

It should be noted that the ITSMC controller designed in this paper has already taken into account uncertainties in system parameters (e.g., variations in tire cornering stiffness *k*_*f*_, *k*_*r*_) during its derivation. Equation (22) decomposes the system dynamics into a nominal part and bounded uncertain parts Δ*f* and Δ*d*, under the assumption that they satisfy the matching condition. This implies that dynamic variations caused by parameter perturbations can be uniformly modeled and compensated through the same control channel. In particular, the introduction of the integral term further enhances the ability to continuously suppress low-frequency parameter uncertainties in real systems, such as slow mass variations and time-varying tire stiffness. Therefore, the designed ITSMC-DYC controller exhibits low theoretical sensitivity to parameter variations that satisfy the matching and boundedness conditions, which provides robustness guarantees for dealing with nonlinear changes in tire characteristics and load fluctuations in practical applications.

### 3.3. Lower-layer torque distribution control system design

The objective of the lower-layer controller is to optimally allocate the total driving torque $T_{x}$ and the additional yaw moment ΔM to the four independent in-wheel motors, based on the dynamic vertical tire loads. This distribution must satisfy both the vehicle’s driving demands and stability requirements while respecting the physical constraints of the actuators and tire-road adhesion limits.

The vertical load on each wheel varies dynamically due to load transfer during longitudinal and lateral accelerations. They are calculated as follows:


$$\begin{array}{c} F_{zfl}=\frac{mgb}{2l}-\frac{ma_{x}h_{g}}{2l}-\frac{ma_{y}bh_{g}}{ll_{w}}\\ F_{zfr}=\frac{mgb}{2l}-\frac{ma_{x}h_{g}}{2l}+\frac{ma_{y}bh_{g}}{ll_{w}}\\ F_{zrl}=\frac{mga}{2l}+\frac{ma_{x}h_{g}}{2l}-\frac{ma_{y}ah_{g}}{ll_{w}}\\ F_{zrr}=\frac{mga}{2l}+\frac{ma_{x}h_{g}}{2l}+\frac{ma_{y}ah_{g}}{ll_{w}} \end{array}$$ (34)

where $F_{zfl},F_{zfr},F_{zrl},F_{zrr}$ are the vertical loads on the left-front, right-front, left-rear, and right-rear wheels, respectively. $a_{x}$ and $a_{y}$ are the longitudinal and lateral accelerations of the vehicle, and $h_{g}$ is the height of the center of gravity (CG).

The core of the distribution strategy is formulated as a Quadratic Programming (QP) problem with the objective of minimizing the tire adhesion utilization ratio η, which is a direct indicator of the vehicle’s stability margin. A smaller η signifies a larger safety margin and a lower risk of tire saturation. For each wheel *i*, the adhesion utilization ratio is defined as:


$$\eta_{i}=\frac{F_{xi}^{2}+F_{yi}^{2}}{{\left(\mu F_{zi}\right)}^{2}},\;i\in \{fl,fr,rl,rr\}$$ (35)

where $F_{xi}$, $F_{yi}$, and $F_{zi}$ are the longitudinal, lateral, and vertical forces on the tire, respectively, and μ is the road adhesion coefficient.

Since the Direct Yaw Moment Control primarily modulates vehicle stability by redistributing longitudinal tire forces, and the lateral force $F_{yi}$ is not directly controlled by the in-wheel motor torques, the optimization objective is simplified to minimize the sum of squared longitudinal force ratios. This leads to the following objective function *J*:


$$\min J=\min\sum_{i}\frac{F_{xi}^{2}}{{\left(\mu F_{zi}\right)}^{2}}=\min\sum_{i}\frac{T_{wi}^{2}}{{\left(\mu F_{zi}R\right)}^{2}},\;i\in \{fl,fr,rl,rr\}$$ (36)

where $T_{wi}$ is the longitudinal drive torque of wheel *i*, and *R* is the effective rolling radius of the wheel.

The torque distribution must satisfy two critical equality constraints that relate the individual wheel torques $T_{wi}$ to the total driving demand $T_{x}$ and the required additional yaw moment ΔM:


$$\left\{ \begin{array}{c} T_{x}=T_{wfl}+T_{wfr}+T_{wrl}+T_{wrr}\\ \Delta M=\frac{d}{2R}(-T_{wfl}+T_{wfr}-T_{wrl}+T_{wrr}) \end{array}\right.$$ (37)

where *d* is the vehicle’s track width.

Let $𝐱=[T_{wfl},T_{wfr},T_{wrl},T_{wrr}{]}^{T}$, ${𝐛}_{eq}=[T_{x},\Delta M{]}^{T}$, and define the equality constraint matrix as: ${𝐀}_{eq}=[\begin{array}{cccc} 1 & 1 & 1 & 1\\ -\frac{d}{2R} & \frac{d}{2R} & -\frac{d}{2R} & \frac{d}{2R} \end{array}]$.

Then, the equality constraint can be expressed as:


$$A_{eq}x=b_{eq}$$ (38)

The solution is bounded by two critical physical limits, forming the inequality constraints:

1 The longitudinal force on each tire must not exceed the available friction force:

$$-\mu F_{zi}R\leq T_{wi}\leq \mu F_{zi}R,\;i\in \{fl,fr,rl,rr\}$$ (39)

2 The torque output of each in-wheel motor is constrained by its peak capability:

$$-T_{\max}\leq T_{wi}\leq T_{\max},\;i\in \{fl,fr,rl,rr\}$$ (40)

where $T_{\max}$ represents the saturation limit of the in-wheel motor, i.e., the peak torque the motor can physically deliver under the current operational conditions. This constraint ensures that the torque allocation demanded by the controller does not exceed the actuator’s hardware capabilities. In this study, $T_{\max}$ is treated as a known, bounded parameter for controller design, typically obtained from the motor’s steady-state torque-speed characteristic map or set as a conservative constant value to guarantee feasibility under all expected driving scenarios.

Combining these two limits, the lower bound ***lb*** and upper bound ***ub*** for the decision variable ***x*** are:


$$\begin{array}{c} \begin{array}{c} lb=\left[\max(-\mu F_{zfl}R,-T_{\max}),\;\max(-\mu F_{zfr}R,-T_{\max}),\;\max(-\mu F_{zrl}R,-T_{\max}),\;\max(-\mu F_{zrr}R,-T_{\max})\right]\\ ub=\left[\min(\mu F_{zfl}R,T_{\max}),\;\min(\mu F_{zfr}R,T_{\max}),\;\min(\mu F_{zrl}R,T_{\max}),\;\min(\mu F_{zrr}R,T_{\max})\right] \end{array} \end{array}$$ (41)

The torque distribution problem is thus formulated as the following standard quadratic programming problem [43]:


$$\left\{ \begin{array}{c} \min J=\min\frac{1}{2}x^{T}Gx+c^{T}x\\ \mathrm{s.t.}\;A_{\mathrm{eq}}x=b_{\mathrm{eq}}\\ \;lb\leq x\leq ub \end{array}\right.$$ (42)

where the Hessian matrix ***G*** is a diagonal matrix defined as $G=diag\left[\frac{\text{2}}{{\left(\mu F_{zfl}R\right)}^{2}},\frac{\text{2}}{{\left(\mu F_{zfr}R\right)}^{2}},\frac{\text{2}}{{\left(\mu F_{zrl}R\right)}^{2}},\frac{\text{2}}{{\left(\mu F_{zrr}R\right)}^{2}}\right]$, and $𝐜={\left[0,0,0,0\right]}^{T}$.

This QP problem is solved in real-time. If an optimal solution is found, the resulting torque vector x* is applied. If the QP solver fails to find a feasible solution, a fallback distribution strategy based on dynamic load distribution is activated to ensure robustness. This strategy allocates the total driving torque equally and distributes the additional yaw moment proportional to the axle loads and track width, as defined below:


$$\left\{ \begin{array}{c} T_{fl}=\frac{F_{zfl}}{F_{zfl}+F_{zfr}+F_{zrl}+F_{zrr}}(\frac{T_{x}}{4}-\frac{4\Delta M}{2l_{w}})R\\ T_{fr}=\frac{F_{zfr}}{F_{zfl}+F_{zfr}+F_{zrl}+F_{zrr}}(\frac{T_{x}}{4}+\frac{4\Delta M}{2l_{w}})R\\ T_{rl}=\frac{F_{zrl}}{F_{zfl}+F_{zfr}+F_{zrl}+F_{zrr}}(\frac{T_{x}}{4}-\frac{4\Delta M}{2l_{w}})R\\ T_{rr}=\frac{F_{zrr}}{F_{zfl}+F_{zfr}+F_{zrl}+F_{zrr}}(\frac{T_{x}}{4}+\frac{4\Delta M}{2l_{w}})R \end{array}\right.$$ (43)

This hierarchical approach—prioritizing optimal adhesion utilization via QP while guaranteeing a feasible, stable output through the fallback strategy—ensures both high performance and essential robustness for the vehicle’s lateral stability control system.

## 4. Simulation and analysis

### 4.1. Simulation and analysis of the state estimation system based on EKF

The simulation verification was conducted in a co-simulation environment integrating MATLAB/Simulink and CarSim. This approach leverages the high-fidelity vehicle dynamics modeling capabilities of CarSim—particularly its accurate tire models, detailed suspension characteristics, and realistic road interaction physics—to provide a more trustworthy validation platform that closely emulates real vehicle behavior. Within this framework, the accuracy of the designed state estimation algorithm was verified.

The road adhesion coefficient is set to 0.85, the low-speed steering wheel angle sine input simulation experiment is performed at a speed of 60 km/h. At the same time, the road is set as a double lane change (DLC) condition, according to ISO3888–1:2018, a good road with a road adhesion coefficient of 0.85 is selected, and a high-speed simulation experiment is carried out at a speed of 120 km/h.

#### 4.1.1. Steering wheel 150-degree sinusoidal input simulation at 60 km/h.

In the steering wheel 150-degree sinusoidal input simulation at 60 km/h, the vehicle operates under moderate lateral excitation, and the system response is dominated by transient steering-induced dynamics. The true vehicle states used for evaluation are obtained from the high-fidelity CarSim model.

**Fig 6:**
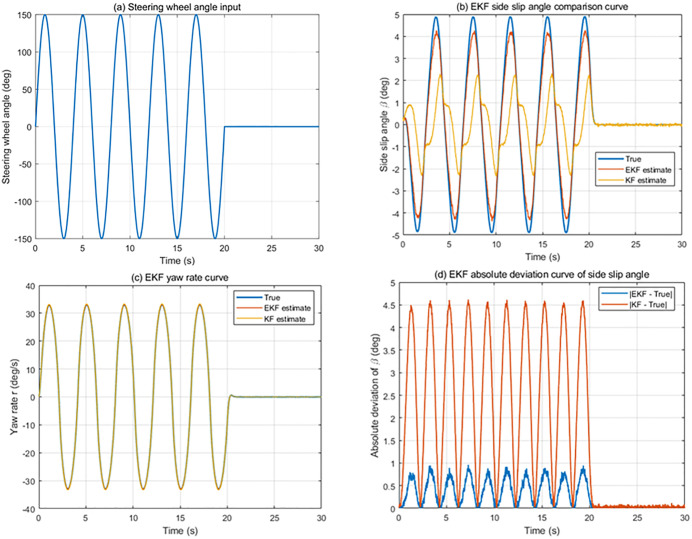
State Estimation System Simulation results of low-speed conditions.

The simulation analysis and comparison results of the vehicle speed of 60 km/h condition are shown in Fig 6. The peak values of each evaluation parameter under 60 km/h conditions was shown in Table 3. As shown in the Fig 6 and Table 3, the EKF is able to rapidly converge to the true yaw rate and sideslip angle at the initial stage of the maneuver. During the sinusoidal steering input, the EKF tracks the variations of both states closely, exhibiting small phase delay and limited estimation overshoot. In contrast, the standard Kalman filter (KF), which is based on a linearized vehicle model, shows noticeable lag and larger transient deviations when the steering input changes direction.

**Table 3:** The peak values of each estimation parameter under 60 km/h condition.

EKF State estimation parameters	Peak deviation (EKF)	Peak deviation (KF)
Side slip angle (deg)	4.59	0.99

These results indicate that, under this moderate-speed transient condition, the EKF provides improved convergence behavior and transient estimation accuracy compared with the KF. The advantage mainly arises from the EKF’s ability to update the system Jacobian online, allowing it to better accommodate the nonlinear characteristics of the lateral vehicle dynamics during steering transients.

#### 4.1.2. DLC simulation at 120 km/h.

The DLC simulation at 120 km/h represents a more challenging scenario, in which the vehicle operates close to its handling limits and tire nonlinearities become more significant. Under this high-speed condition, the mismatch between the simplified observer model and the actual vehicle dynamics is amplified.

**Fig 7:**
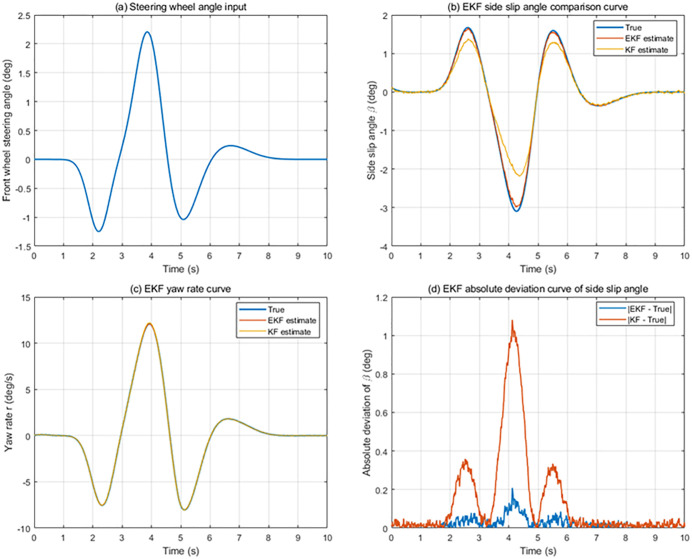
State Estimation System Simulation results of high-speed conditions.

The simulation analysis and comparison results of the vehicle speed of 120 km/h at DLC condition is shown in Fig 7. The peak values of each evaluation parameter under 120 km/h condition are shown in Table 4. From the simulation results, it can be observed that the estimation errors of both the EKF and the KF increase compared with the low-speed case. However, the EKF maintains stable convergence and bounded estimation errors throughout the maneuver. The yaw rate and sideslip angle estimates produced by the EKF remain consistent with the CarSim reference, even during rapid lateral load transfer and aggressive steering inputs.

**Table 4:** The peak values of each estimation parameter under 120 km/h condition.

EKF State estimation parameters	Peak deviation (EKF)	Peak deviation (KF)
Side slip angle (deg)	1.09	0.21

In contrast, the KF performance deteriorates in this scenario. Larger estimation deviations and increased phase lag can be observed, particularly during the peak lateral acceleration intervals of the DLC maneuver. This behavior reflects the limited capability of the linear KF to capture strong nonlinear tire forces at high speed.

Overall, the DLC results demonstrate that the EKF exhibits superior robustness and reliability under high-speed and highly nonlinear conditions, making it more suitable for providing accurate state feedback for lateral stability control in extreme driving scenarios.

### 4.2. Simulation and analysis of the stability control system based on ITSMC

The simulation verification was conducted in a co-simulation environment integrating MATLAB/Simulink and CarSim, leveraging CarSim’s high-fidelity vehicle dynamics model to ensure realistic validation. Within this framework, the effectiveness of the proposed ITSMC controller was evaluated and compared against both the baseline of no control and a conventional SMC strategy.

The ice and snow road with a road adhesion coefficient of 0.1 is set, the low-speed steering wheel angle sine input simulation experiment is performed at a speed of 60 km/h. At the same time, the road is set as a double lane change (DLC) condition, a bad road with a road adhesion coefficient of 0.2 is selected, and a high-speed simulation experiment is carried out at a speed of 120 km/h. The peak values of each evaluation parameter under the 60 km/h condition are shown in Table 5. The absolute value of parameter peak with ITSMC controller or without control under 120 km/h condition are shown in Table 6.

**Table 5:** The absolute value of parameter peak with ITSMC control or without control.

Absolute value of parameter peak	No control	SMC control	ITSMC control
Side slip angel (deg)	64.55	1.698	1.109
Yaw rate (deg/s)	15.75	13.72	13.17

**Table 6:** The absolute value of parameter peak with ITSMC control or without control.

Absolute value of parameter peak	No control	SMC control	ITSMC control
Side slip angel (deg)	2.891	2.914	2.078
Yaw rate (deg/s)	90.11	8.415	5.106

#### 4.2.1. Steering wheel 150-degree sinusoidal input simulation at 60 km/h.

**Fig 8:**
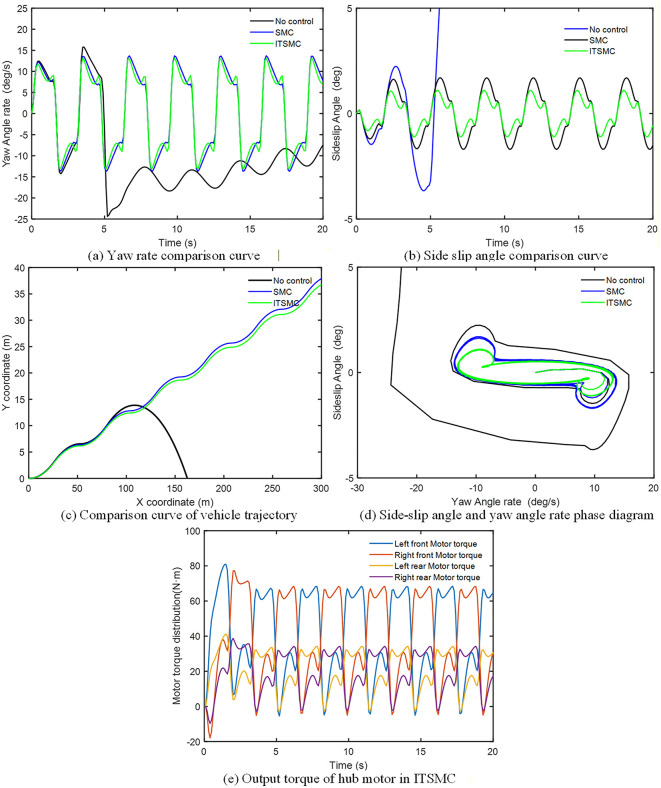
Stability Control System Simulation results of low-speed conditions.

The simulation analysis and comparison results of the vehicle speed of 60 km/h condition are shown in Fig 8. As shown in Fig 8a, b and Table 5, the yaw rate and the sideslip angle under uncontrolled conditions begin to oscillate drastically after about 5 s, with the yaw rate peaking at 65.55 °/s and the sideslip angle reaching 15.75 °. Both exceed the stability threshold, leading to loss of vehicle control. In contrast, the two control methods effectively suppress wheel slip. Compared with sliding mode control, integral terminal sliding mode control provides more precise restraint of the yaw rate overshoot and keeps the sideslip angle within a stable range of 1.5 °.

It is crucial to emphasize that these simulations are based on the high-fidelity CarSim model, whose Magic Formula tire model realistically captures the complex nonlinear and time-varying relationships between tire force and slip ratio, vertical load, and road conditions. During the aggressive sinusoidal steering maneuver on the set low-adhesion road, the effective tire cornering stiffness undergoes significant and continuous variations. This creates an inherent and severe “parameter uncertainty” environment for testing the controllers. Within this environment, the phase trajectory envelope under ITSMC (Fig 8d) is smaller and more convergent than that under SMC, and the path tracking deviation is also smaller (Fig 8c). This directly demonstrates that ITSMC exhibits lower sensitivity and stronger robustness to parameter variations induced by the actual nonlinear tire dynamics. Fig 8e further illustrates that under extreme conditions, differential braking/driving of the left and right wheels is necessary to generate the required yaw moment. The torque distribution strategy adopted here better utilizes the traction of tires with higher axle loads while improving the stability margin of tires with lower axle loads. As a result, the output torque of the front axle hub motor consistently exceeds that of the rear axle.

#### 4.2.2. DLC simulation at 120 km/h.

**Fig 9:**
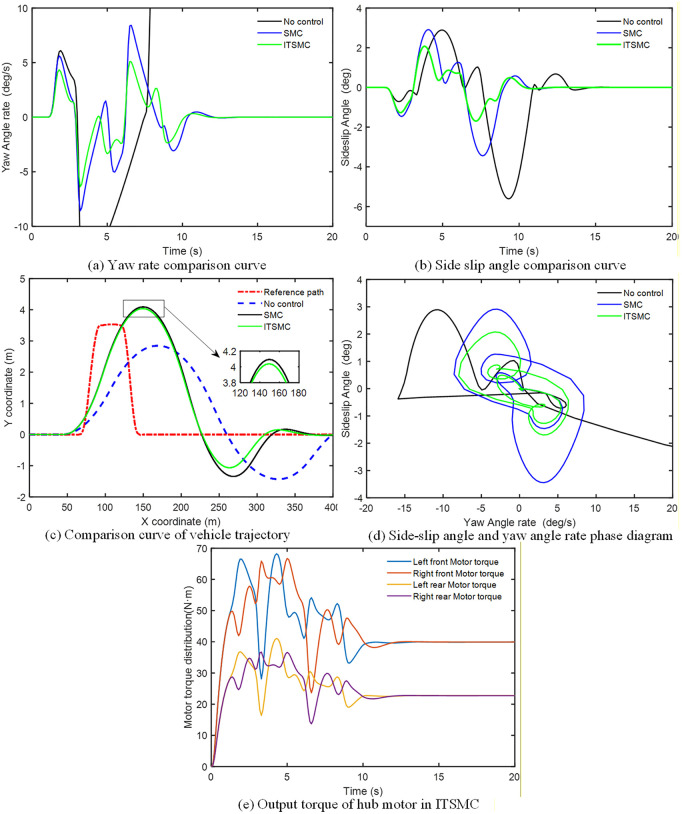
Stability Control System Simulation results of high-speed conditions.

The simulation analysis and comparison results of the vehicle speed of 120 km/h at DLC condition are shown in Fig 9. As shown in Fig 9a, b and Table 6, under uncontrolled conditions, the yaw rate exhibits severe oscillations after about 5 s, with its peak reaching 90.11 °/s, while the peak sideslip angle reaches 2.891°. Both exceed the stability threshold, leading to vehicle instability. In contrast, both control methods effectively suppress wheel slip. Compared with sliding mode control, integral terminal sliding mode control provides more precise control over the yaw rate overshoot and confines the sideslip angle within a stable range of 2 °.

To directly assess the controller’s sensitivity to parameter uncertainties, the severity of this simulation scenario must be highlighted: a double lane change maneuver at 120 km/h on a road with an adhesion coefficient of only 0.2. The tire forces (particularly lateral forces) generated by the high-fidelity CarSim tire model under these conditions are strongly nonlinear, time-varying functions of slip angle and vertical load. This is equivalent to injecting continuous and severe variations in effective tire cornering stiffness into the control system. Within this “field of parameter uncertainty,” the quantitative comparison in Table 6 is revealing: the peak yaw rate under ITSMC control (5.106 deg/s) is significantly lower than that under SMC (8.415 deg/s), representing a reduction of approximately 39%. This performance gap visually reflects the lower sensitivity of ITSMC to parameter uncertainties arising from the nonlinear, time-varying characteristics of the tires. Fig 9c indicates that the uncontrolled vehicle deviates from the reference path and loses control at approximately 150 m and 300 m, respectively. Both control methods, however, enable the vehicle to maintain normal driving on low adhesion road surfaces and eventually return to the reference path. Moreover, at the 150 m mark, the path tracking error under ITSMC is smaller than that under SMC. The phase portrait in Fig 9d shows that the convergence region of ITSMC is smaller than that of SMC, demonstrating the superior control performance of ITSMC. Fig 9e further illustrates that under extreme conditions, yaw moment control requires braking one wheel while driving the other. The torque distribution strategy adopted in this work optimizes the utilization of the adhesion capacity of tires with higher axle loads while enhancing the stability margin of tires with lower axle loads. Consequently, the output torque of the front axle hub motor remains consistently higher than that of the rear axle.

## 5. Hardware-in-the-loop (HIL) test analysis

To better validate the availability of the built ITSMC controller, a semi-physical simulation HIL test platform was established to test the designed control strategy.

**Fig 10:**
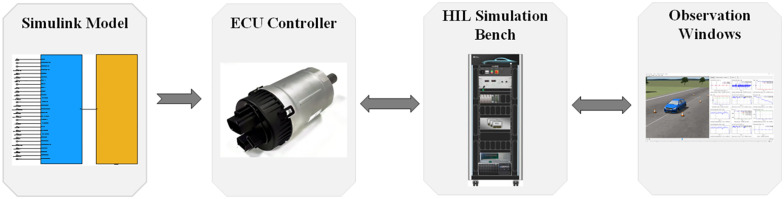
The HIL test system block diagram.

The basic process of HIL testing mainly includes the following contents, First, it is necessary to convert the designed Simulink model program into code and write it to the controller. Then, through the input/output interface, the controller and hardware are linked to the loop simulation platform. Finally, the test results are observed by the observer. The HIL test system block diagram is shown in Fig 10.

**Fig 11:**
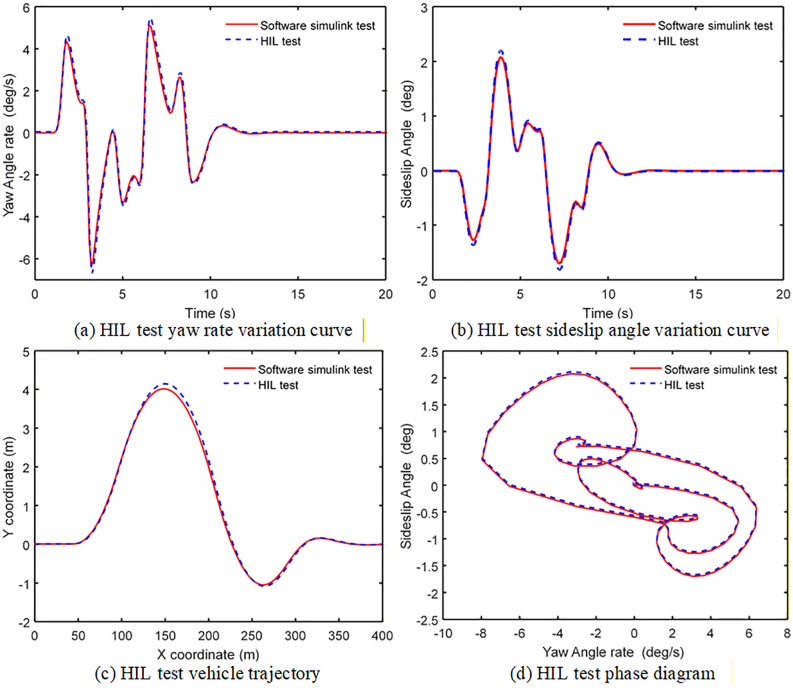
The HIL test and simulation comparison results.

The HIL simulation test was carried out only in the DLC condition of 120 km/h and road adhesion coefficient 0.2. The HIL test and simulation comparison results are shown in Fig 11. The test and simulation results are shown in Table 7.

**Table 7:** Parameter curve peak.

Control method	Yaw rate(deg/s)	Side slip angle(deg)
Software simulation test	5.016	2.078
HIL test	5.125	2.193

It can be seen from Fig 11 and Table 7, that the results of HIL test and software simulation are slightly different. The maxi-mum deviation of yaw rate comparison curve is 2.12%, and the maximum deviation of sideslip angle comparison curve is 5.24%, the error values of the two states are within 5%. At the same time, the vehicle trajectory curve, the sideslip angle and the yaw rate phase curve are also basically coincident, which indicates that the ITSMC controller has good controlling effect and adaptability.

It should be noted that several practical issues may arise in real-world implementation. The performance of the EKF may be influenced by sensor noise, bias, and model uncertainties, which require appropriate noise covariance tuning in practical applications. In addition, the proposed control strategy involves state estimation, yaw moment control, and torque allocation, and therefore computational delay and real-time feasibility should be carefully considered for embedded electronic control units. Although each module is designed with moderate computational complexity, discretization effects and communication delays may affect control performance under aggressive maneuvers. These issues can be mitigated through efficient implementation, proper sampling rate selection, and controller parameter tuning.

## 6. Conclusions

(1) An EKF-based vehicle state estimation algorithm was developed to provide accurate and smooth estimates of the sideslip angle and yaw rate under highly nonlinear and time-varying driving conditions. Compared with observer-based or particle filtering approaches commonly reported in the literature, the proposed EKF scheme achieves a better balance between estimation accuracy, computational efficiency, and real-time feasibility, making it more suitable for integration with nonlinear stability controllers.
(2) An integral terminal sliding mode control–based direct yaw moment controller was designed to enhance vehicle lateral stability under parameter uncertainties and external disturbances. In contrast to conventional sliding mode–based DYC strategies, the proposed ITSMC effectively suppresses control chattering while ensuring finite-time convergence, resulting in improved robustness and smoother yaw-moment generation under extreme low-adhesion and high-speed conditions.
(3) A hierarchical control architecture integrating EKF-based state estimation, ITSMC-based yaw-moment control, and an optimal control–based wheel torque allocation strategy was established and validated through co-simulation and hardware-in-the-loop tests. The results demonstrate that the proposed method achieves superior lateral stability performance, reduced yaw rate overshoot, and improved trajectory tracking accuracy compared with traditional DYC approaches, highlighting its practical advantages for distributed drive electric vehicles operating near handling limits.
